# Decision Support System for Emergencies in Microgrids

**DOI:** 10.3390/s22239457

**Published:** 2022-12-03

**Authors:** Maria Fotopoulou, Dimitrios Rakopoulos, Stefanos Petridis

**Affiliations:** Centre for Research and Technology Hellas, Chemical Process and Energy Resources Institute, 52 Egialias Str., GR-15125 Athens, Greece

**Keywords:** microgrid, decision support system, emergency, optimization, energy management system, autonomy, blackout, black start, islanded mode, renewables, batteries

## Abstract

The usual operation of a microgrid (MG) may often be challenged by emergencies related to extreme weather conditions and technical issues. As a result, the operator often needs to adapt the MG’s management by either: (i) excluding disconnected components, (ii) switching to islanded mode or (iii) performing a black start, which is required in case of a blackout, followed by either direct reconnection to the main grid or islanded operation. The purpose of this paper is to present an optimal Decision Support System (DSS) that assists the MG’s operator in all the main possible sorts of emergencies, thus providing an inclusive solution. The objective of the optimizer, developed in Pyomo, is to maximize the autonomy of the MG, prioritizing its renewable production. Therefore, the DSS is in line with the purpose of the ongoing energy transition. Furthermore, it is capable of taking into account multiple sorts of Distributed Energy Resources (DER), including Renewable Energy Sources (RES), Battery Energy Storage Systems (BESS)—which can only be charged with renewable energy—and local, fuel-based generators. The proposed DSS is applied in a number of emergencies considering grid-forming and grid-following mode, in order to highlight its effectiveness and is verified with the use of PowerFactory, DIgSILENT.

## 1. Introduction

Microgrids (MGs) are self-sufficient intelligent grids that can accommodate a plethora of Distributed Energy Resources (DER), such as Renewable Energy Sources (RES) [1,2,3,4], and also integrate storage units [5,6,7,8]. Their self-sufficiency stems from the ability to operate either in association with a power grid (grid-following mode) or independently (islanded mode) [9,10]. In addition, they contain intelligent capabilities such as Decision Support Systems (DSS) that can operate independently or guide the system’s operator [11,12,13]. MGs often encounter emergencies related to technical issues or extreme weather conditions [14]. Therefore, the system’s DSS needs to be able to assess the current situation of the grid and assist the operator in making certain modifications in the MG’s management for emergency circumstances, until it returns to its regular operating condition.

**Figure 1:**
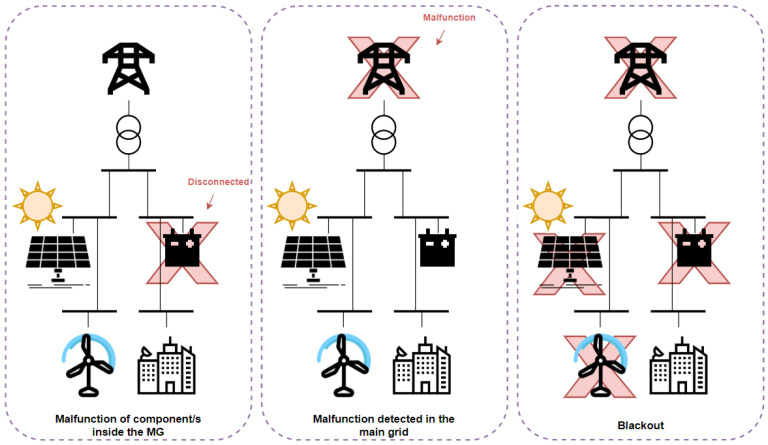
Main sorts of emergencies.

The emergency scenarios encountered by a MG and solved by a DSS algorithm, found in the literature, can be classified into three main categories and are shown in Figure 1: (i) malfunction of components inside the MG, where one or multiple components inside the MG do not operate properly or are completely disconnected while it is operating in grid-following mode (connected to the main grid) [15], (ii) malfunction detected in the main grid, where an issue in the main grid feeding the MG is experienced; hence, the MG is required to function in islanded mode, disconnected from the main grid [16] and (iii) blackout, which is the most severe type of emergency that can be encountered by a MG, where the main grid and most MG components are out of service. In this situation, the MG operator needs to perform a black start which is a valuable ancillary service for the main grid operator [17].

In the literature, extensive research has been conducted to address all three types of emergencies mentioned, providing useful tools/algorithms that could be integrated in a DSS that aims to handle emergencies in MGs. Addressing the first type of emergency that such a DSS should be able to solve, which is due to MG components’ malfunctions, the authors of [15] propose an algorithm for handling emergencies in MGs with high RES penetration and assess the effect of the disconnection of various supplies. Furthermore, in [18], an emergency energy management method based on mixed-integer nonlinear programming (MINLP) is proposed. The authors of [19] present a mathematical programming based on a MG formation method that approximates the MINLP problem with multiple iterations of mixed-integer linear programming (MILP) problems.

For MGs operating in islanded mode (which is the second type of emergency that an emergency-related DSS would often need to overcome), in [16], two control strategies are evaluated for when the power from the main grid is lost, one utilizing a single voltage source inverter as a voltage reference and another employing multiple voltage source inverters. The authors of [20] propose a systematic methodology for optimal load selection and control in situations of limited to no available power. A methodology for distributed secondary control in islanded MGs is proposed in [21]. Last but not least, a rule-based algorithm for managing unplanned islanding events in MGs with storage units and DER is shown in [22].

Finally, a black-start operation, which is the third type of emergency that needs to be taken into account by an emergency-related DSS (as it occurs frequently), is mentioned in the literature as well. In [23], a black-start strategy is presented for MGs containing photovoltaics (PVs) and hybrid storage systems. A sequential service restoration method is proposed by [24], which also employs the frequency response of a single master operation MG to calculate the safest time intervals between restoration steps. A sequential methodology is proposed in [25] as well, where the framework developed generates restoration sequences to coordinate the distributed generation (DG) operation. In [26], the authors present a sequential method for distribution system restoration, exploiting the multi-MG concept. The authors of [27,28,29], on the other hand, propose methodologies focusing on parallel restoration. To conclude, in [30], the dynamic phenomena occurring during a MG restoration are presented.

A unified methodology that tackles all possible emergencies faced by MGs is not found in the open literature. The purpose of this paper is to propose a DSS framework for solving all the previously mentioned types of emergencies, expanding the purpose of the aforementioned works. The proposed DSS framework assesses the type of emergency taking place and automatically calibrates its behavior to facilitate the MG’s needs, resulting in a unified solution for all types of emergencies. For this purpose, it is based on a MILP optimizer that adapts depending on the type of emergency and the timescale and is able to control a mix of high DER penetration and storage systems. The objective function presented aims to maximize the system’s autonomy, focusing on the RES production, being in tune with the requirements of the emerging energy transition. The DSS is developed in Pyomo [31] and applied on three different emergency scenarios to showcase its performance, flexibility and efficiency, and it is verified with the use of PowerFactory, DIgSILENT [32].

## 2. Methodology

**Figure 2:**
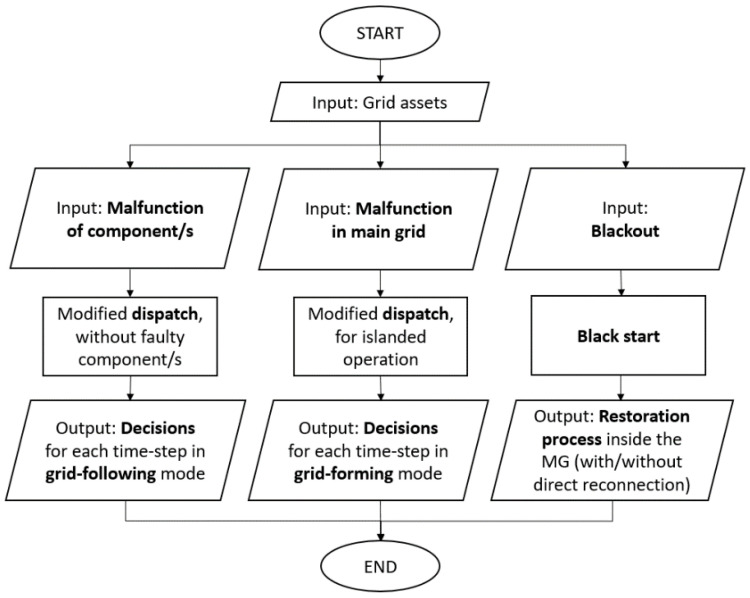
Main decision support flow chart.

**Figure 3:**
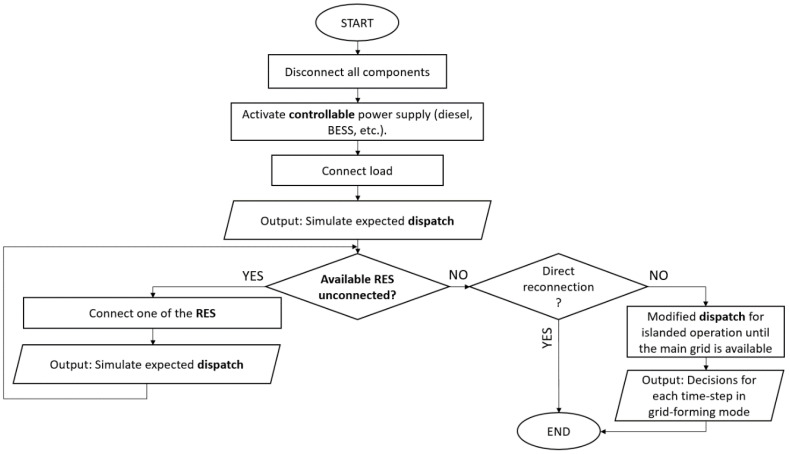
Black start flow chart.

The flow chart of the proposed DSS is presented in Figure 2. More specifically, if the emergency is related to part of the MG, then the DSS modifies the optimizer so that the faulty component/s are excluded and provides the optimal dispatch considering the remaining components while maintaining grid-following mode, in which the main grid dictates the voltage of the MG. On the other hand, if the emergency is related to a malfunction in the main grid which is identified on time by the monitoring/safety system, the DSS modifies the optimizer so that the MG can operate on island mode. This means that it will operate disconnected from the main grid, in grid-forming mode, where the MG follows the voltage of one or more of its active components, supported by the respective specialized energy dispatch, until it is safe to reconnect to the main grid. Finally, if there is a blackout, the DSS performs a black start, the flow chart of which is presented in Figure 3. This is considered to be the most complex and challenging emergency and shall be explained thoroughly below.

In more detail, as presented in Figure 3, when a blackout occurs, the first step would be to disconnect all of the components. Afterwards, the controllable power supply units, such as diesel generators, storage, etc., are the first ones to be activated, followed by the load. At this point, the DSS modifies the optimizer in order to include only the aforementioned components and the time-step needs to be adjusted to the needs of the black start, i.e., from one or half an hour (which is the usual case) it needs to be reduced to a few seconds/minutes (depending on the requirements of each component). Having provided the optimal decisions, the next step is to gradually reconnect the RES of the MG, modifying the optimizer accordingly every time that the loop is repeated (once for each RES). In the end, all controllable and non-controllable units are reconnected and feed the load. At this point, the DSS checks if the main grid is ready for reconnection. It should be highlighted that there are many cases when the main grid may not be ready for reconnection for a long time interval, e.g., a few hours, due to extreme weather conditions that pose great challenges to repairing actions. In this case, the MG needs to operate islanded for as long as required, as it would in the second sort of emergencies, before it is reconnected to the main grid, switching from grid-forming to grid-following mode.

All of the rules and functionalities described in the theory above are coded in the proposed DSS. Consequently, it is evident that, at some point, all types of emergencies require a modified dispatch that provides the optimal decisions regarding the available network components. This translates to developing a flexible optimizer that can operate regardless of the number of available network components, for any time interval and time-step. Therefore, the core of the algorithm is an abstract MILP optimizer, applicable on MGs that include any number [0, 1, 2…) of the following assets: RES, generators and Battery Energy Storage Systems (BESS). The decision variables include the power charged to/discharged from each BESS, the production of the generators and RES curtailment, if required.

The objective function of the optimizer is presented in (1). Its aim is to maximize the autonomy of the MG, prioritizing RES-based energy, following the trend of the ongoing energy transition. It is highlighted that the optimizer is constructed in a way that the BESS can only be charged by the RES.

More specifically, the use of the RES production has zero weight and, therefore, is not mentioned in the objective function, thus being the optimal/first choice out of all energy sources. The second preferable supply units are the smart grid’s BESS (one or more, if existing). Their contribution is considered to be environmentally friendly as they can be charged only by the RES and enhance the autonomy of the MG since they are flexible. Therefore, the energy discharged from each BESS at each time-step, t, $D_{b,t}$, has the lowest weight, $w_{1,b}$, in the objective function. The next preferable supply units are the local fuel-based generators (if any), e.g., diesel generators, $G_{g,t}$, the production of which has a higher weight than the BESS, $w_{2,g}$. The least preferable source is the main grid, the contribution, $Img_{t}$, of which has the highest weight out of all sources, $w_{3}$, as opposing the grid autonomy. Finally, in order to ensure the maximum use of the RES production and the maximum possible autonomy, the curtailed energy from the RES, $R_{n,t}^{curt}$, has the highest weight of all, $w_{4,n}$.

(1) $$\min F=\underset{t}{\sum }(\underset{b}{\sum }w_{1,b}D_{b,t}+\underset{g}{\sum }w_{2,g}G_{g,t}+w_{3}Img_{t}+\underset{n}{\sum }w_{4,n}R_{n,t}^{curt})$$

Utilizing the network’s monitoring data, the DSS framework is able to evaluate the type of emergency that has occurred and dynamically adjust its optimizer parameters. Of course, since the developed DSS needs to be flexible, in order to be able to deal with all possible emergency cases, if the emergency is related to islanded operation or a black start, the contribution of the main grid, $Img_{t}$, is automatically removed from the objective function and the constraints of the optimizer. Furthermore, if a component either: (a) does not exist in the distribution network (e.g., a distribution network may have a RES and BESS but no generators), (b) is out of service, or (c) has not been activated yet (e.g., during the gradual reconnection of components in a black start, presented in Figure 3), then it is automatically removed from the objective function and the respective constraints of the optimizer.

Each BESS is modeled using (2)–(6). The energy balance of each BESS is represented by (2), where $S_{b,t}$ is the stored energy of each BESS, b, at time-step t. $S_{b,t-1}^{\;}$ is their former stored energy, $C_{b,t}$ is the energy charged to each BESS at each time-step t and $\eta_{b}$ is the efficiency. Furthermore, (3) denotes the capacity limitations of each BESS, where $S_{b}^{min}$ is the minimum and $S_{b}^{max}$ is the maximum value. Constraints (4) and (5) denote the maximum energy that can be discharged from (or charged to) a BESS at each time-step, respectively, where $D_{b}^{max}$ and $C_{b}^{max}$ are the maximum values and $u_{b,t}^{ch}$, $u_{b,t}^{dch}$ are the binary variables that are activated if the BESS is charged or discharged, respectively. Constraint (6) means that a BESS cannot be charged and discharged simultaneously.

(2) $$S_{b,t}=S_{b,t-1}^{\;}+C_{b,t}\eta_{b}-D_{b,t}/\eta_{b}$$

(3) $$S_{b}^{min}\leq S_{b,t}\leq S_{b}^{max}$$

(4) $$D_{b,t}\leq D_{b}^{max}u_{b,t}^{dch}$$

(5) $$C_{b,t}\leq C_{b}^{max}u_{b,t}^{ch}$$

(6) $$u_{b,t}^{dch}+u_{b,t}^{ch}=1$$

The use of each RES is modeled by (7), where $R_{n,t}$ is the RES production of the n-th plant at time-step t and can be either used in the distribution network, $R_{n,t}^{use}$, or curtailed, $R_{n,t}^{curt}$. It should be noted that if the grid restoration process does not require islanded operation (this happens in the first of the aforementioned emergencies, where the distribution network remains in grid-following mode), then it is possible for the amount of energy that would otherwise be curtailed to be injected to the main grid, as an alternative. This reverse flow of energy might be useful to the main grid operator, especially when the main grid faces issues related to a drop in voltage levels from the nominal values.

(7) $$R_{n,t}=R_{n,t}^{use}+R_{n,t}^{curt}$$

The nominal value of each generator, $G_{g}^{max}$, provided that the MG includes generators, is taken into account by (8).

(8) $$G_{g,t}\leq G_{g}^{max}$$

The energy balance of the MG at each time-step t is modeled by (9), where $L_{i,t}$ is the load of the i-th node.

(9) $$\underset{i}{\sum }L_{i,t}+\underset{b}{\sum }C_{b,t}=\underset{b}{\sum }D_{b,t}+\underset{g}{\sum }G_{g,t}+\underset{n}{\sum }R_{n,t}^{use}+Img_{t}$$

The aforementioned problem formulation can be used for all sorts of emergencies since it is abstract. For example, it can be applied on a 3-h islanded operation emergency with hourly time-steps. In addition, it can be applied for single time-steps for every single stage of a black start, where the duration of each time-step is much lower, e.g., 1 min, 0.5 min, etc., [33]. This flexibility provides the DSS with a uniform approach.

The DSS is developed in Python and the optimizer is developed using Pyomo [31]. The selected solver is Bonmin [34], due to its wide implementation in dispatch optimization problems. The proposed DSS is verified with the use of PowerFactory, DIgSILENT. It is noted that a few assumptions/simplifications have been made in the development of the optimizer. For example, the parameters of the distribution lines, the topology and the voltage of each node of the MG are not taken into account. The aforementioned modifications turn the problem from MINLP into MILP. Therefore, the algorithm converges faster, which is a major requirement during emergencies. Moreover, as verified by dedicated simulations with PowerFactory, DIgSILENT, since MGs only contain few nodes and their area expansion is limited, these modifications do not affect the accuracy of the results significantly, i.e., less than 1%. It needs to be mentioned that the operation of this DSS framework is limited to regular MGs that do not include a large amount of nodes. In a scenario of a very large MG, the aforementioned assumptions and problem modifications would degrade the DSS framework’s performance.

## 3. Use Cases

**Figure 4:**
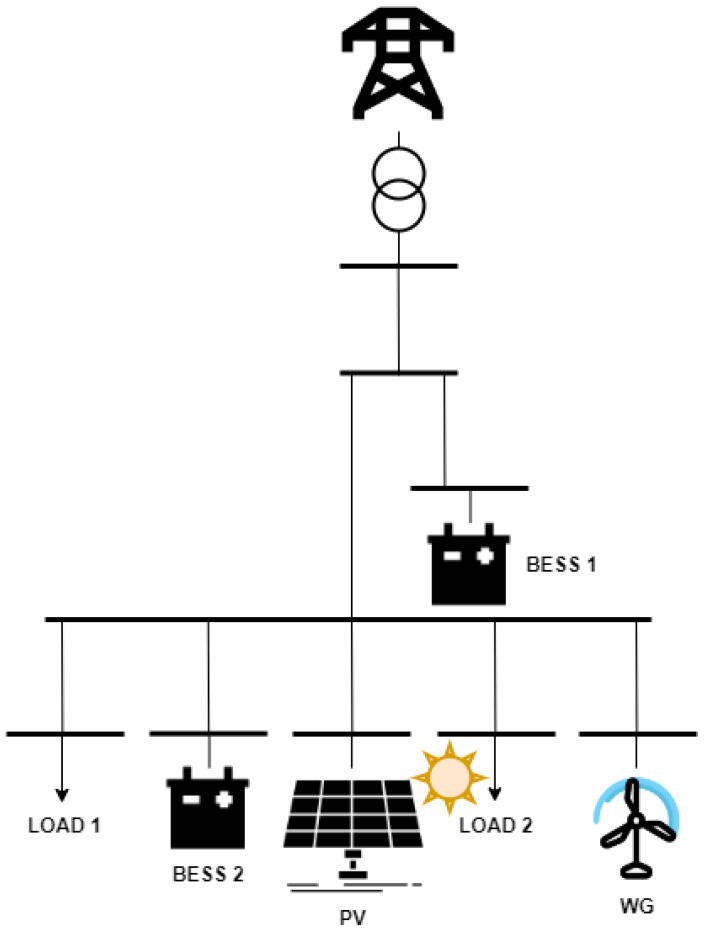
Graphical representation of CIEMAT’s MG.

The proposed DSS is hypothetically applied on the MG of CIEMAT [35], which is located in Soria, Spain, presented in Figure 4 and in the Appendix A. This MG is a key demonstrator of the TIGON project [36], which promotes the efficient use of Direct Current (DC)-based RES and BESS in distribution grids and MGs in both Medium Voltage (MV) and Low Voltage (LV) levels, in the context of which this DSS is developed. The MG includes two kinds of RES, i.e., Photovoltaics (PVs) and a Wind Generator (WG), as presented in Table 1. The installed power of the PVs is justified by the size of the installed load. The WG, the size of which is close to half of the installed load, is added in order to have a more diverse RES production profile, since (in contrast to the PVs) they also produce energy during night hours. In addition, the MG is equipped with two BESS (which is more complex than having one BESS, thus highlighting the ability of the optimizer to deal with multiple decision variables, unlike respective rule-based algorithms). The first BESS, i.e., BESS 1, which is larger than the second one, is the main controllable power supply unit in case of an emergency, due to its size. This means that the assigned weight in the objective function (1), $w_{1,1}$, is slightly lower than the respective weight of the second BESS, $w_{1,2}$, in order to be selected by the optimizer. The nominal values of the two BESS are corresponding to the size of the RES components and the load requirements.

**Figure 5:**
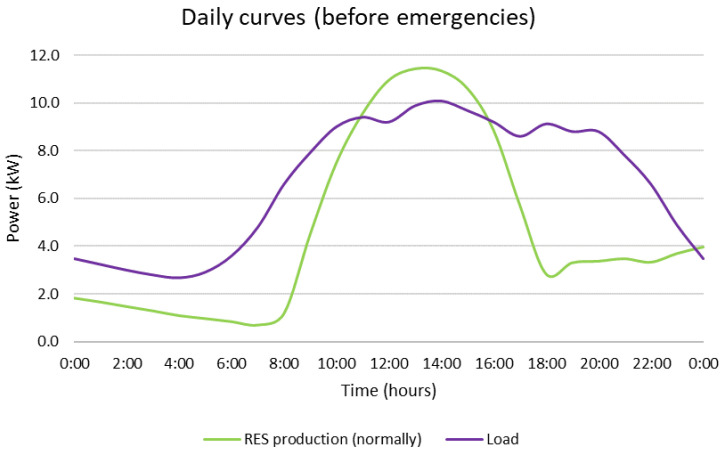
Profile of the simulated day without the emergencies.

The daily curves of the simulated day are presented in Figure 5. The load is assumed to follow a mixed daily profile, including residential, commercial and industrial parts, provided by the benchmark systems of [37]. The RES production profile is derived from [38] and represents a representative day of the year for the location of the CIEMAT MG, Spain. The daily curves of Figure 5 showcase the suitability of this MG as a test grid for the developed framework as the rich energy mix and the alternation between the production and demand profile render the implementation of an optimizer-based DSS a necessity for the efficient management of all resources.

In order to highlight the flexibility of the proposed DSS, one emergency of each sort is simulated, as presented in Table 2. More specifically, the first emergency under study, i.e., Case 1, is the temporary disconnection of the MG’s second BESS from 13:00 up to 16:00. The emergency lasts for three hours, which is challenging, taking into consideration that the acceptable values of the System Average Interruption Duration Index (SAIDI) and the Customer Average Interruption Duration Index (CAIDI) are 1.5 and 1.36 h, respectively [39]. The selected time interval is based on the worst-case scenario principle and justified by the peak values of both the RES and demand curves, which occur during noon. The duration of each time-step for Case 1 is equal to one hour. The second emergency under study, i.e., Case 2, is an issue related to the main grid which requires the islanded operation of the MG for six hours, from 13:00 until 19:00. The selected time interval not only includes the peak of the RES and the peak of the load, but also a time interval during which the load is higher than the RES production. As in Case 1, the duration of each time-step for Case 2 is equal to one hour. Finally, the third emergency under study, i.e., Case 3, denotes an extremely challenging scenario, where a black start needs to be performed at 18:00, when the difference between the demand and the RES production is the highest, and the MG needs to operate islanded for the following three hours until it is safe to reconnect to the main grid. For the purpose of the simulation, the duration of each time-step of the black start is considered to be equal to one minute [33].

## 4. Results

The results which the developed DSS provided for each use case are presented in the following sub-sections.

### 4.1. Results of Case 1

**Figure 6:**
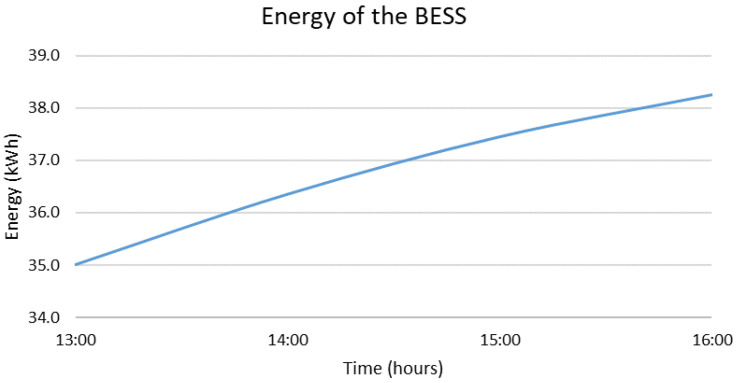
Energy stored in BESS 1 if BESS 2 is disconnected from 13:00 to 16:00.

**Figure 7:**
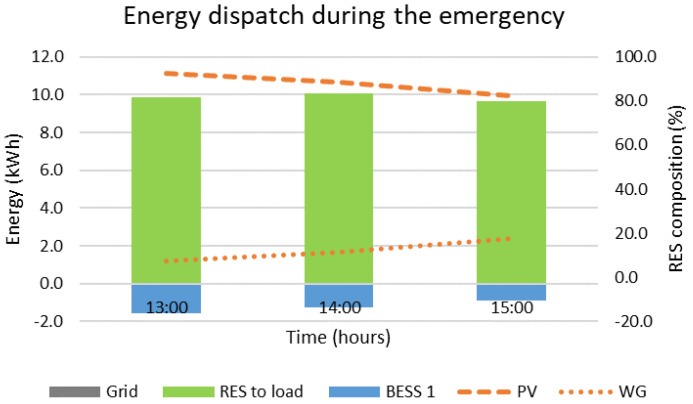
Energy dispatch if BESS 2 is disconnected from 13:00 to 16:00.

In case the MG operates in grid-following mode and the second BESS needs to be disconnected from 13:00 to 16:00, the optimal decisions provided by the DSS are presented in Figure 6 and Figure 7. In more detail, Figure 6 presents the energy stored in the main BESS (BESS 1) during the emergency. It is noted that during this time interval the RES production is higher than the demand. The surplus production that would normally be absorbed by both BESS of the MG is now absorbed only by BESS 1. This results to a 9.3% increase in the state of charge. Since this does not challenge the limits of the BESS, no RES curtailments are required. The energy dispatch during the emergency is presented in Figure 7. The hourly contribution of the BESS is negative since it is charged (not discharged). Throughout the emergency, the load is covered by the PVs and the WG and no energy from the main grid is required. Overall, it can be concluded that this sort of emergency has a relatively low impact on the overall operation of the MG, even though its duration is equal to twice the acceptable values of SAIDI found in the literature and the selected time interval includes the load’s peak. The main related values are also summarized in Table 3 at the end of this section. 

The results highlight the orientation of the optimizer toward autonomy and RES prioritization. For example, a DSS with a different objective function, e.g., cost minimization, distribution losses minimization, etc., would probably result in deriving different decisions. In more detail, if, for example, the purpose of the MG was to assist the main grid operator during noon hours by minimizing the main grid’s voltage deviations that often occur at that time interval due to the high energy consumption, it is possible that the extra RES energy would not be stored in BESS 1 but injected to the main grid. Furthermore, if the MG was not equipped with an optimizer-based DSS at all, then human-observer decisions could be different from the proposed ones because there would be no standard, model-based approach. This could result in a decision of unnecessary RES curtailments or charging BESS 1 with more/less energy than the optimal value. Of course, the same observation applies to all sorts of emergencies.

### 4.2. Results of Case 2

**Figure 8:**
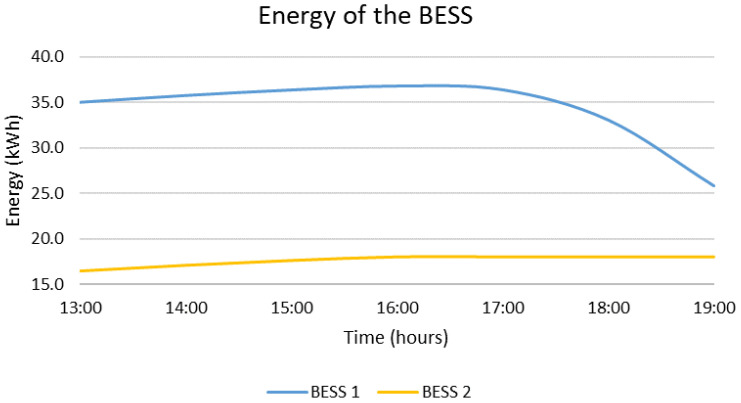
Energy stored in each BESS for islanded operation from 13:00 to 19:00.

**Figure 9:**
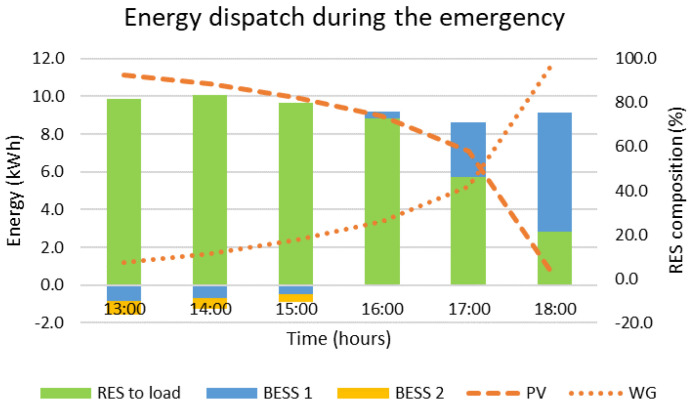
Energy dispatch for islanded operation from 13:00 to 19:00.

In case the MG needs to be disconnected from the main grid from 13:00 to 19:00, the optimal decisions provided by the DSS are presented in Figure 8 and Figure 9. According to the results, the two BESS are charged with the surplus RES production for the first three hours of the emergency. Afterwards, the load exceeds the RES production, which is expected due to the fact that most of the production comes from the PVs. As a result, the main BESS is discharged while the second BESS remains as is. From the beginning up to the end of the emergency, the state of charge of the main BESS is reduced by 26.2% and the state of charge of the second BESS is increased by 9.0%. During the emergency, no RES curtailments are required. Furthermore, as presented in Figure 9, the load is covered mostly by the RES, i.e., 83.2%, and partly by the main BESS, i.e., 16.8%. The main results are summarized in Table 3 at the end of this section.

### 4.3. Results of Case 3

**Figure 10:**
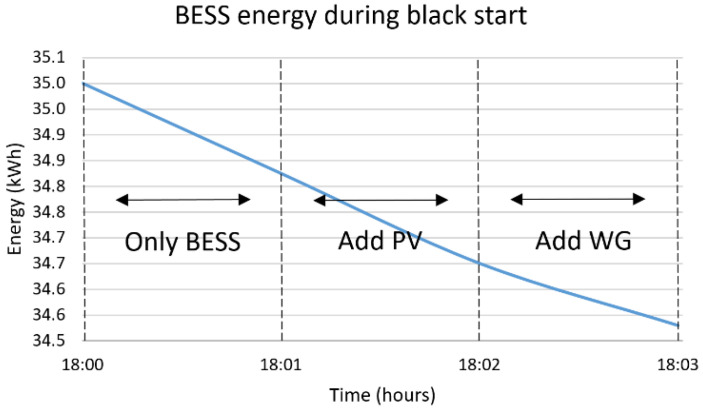
Energy stored in BESS 1 for a black start at 18:00.

**Figure 11:**
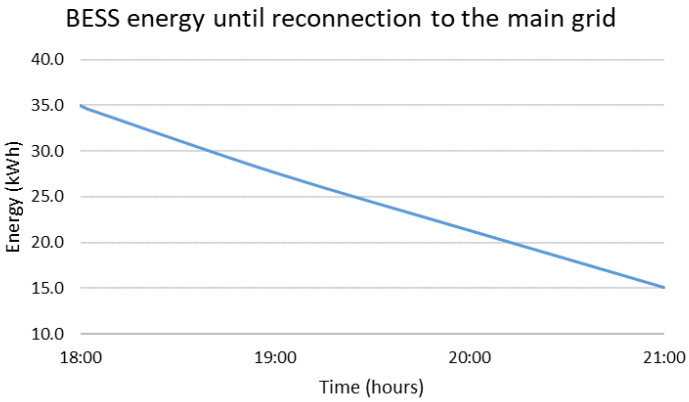
Energy stored in BESS 1 for a black start at 18:00 followed by islanded operation until 21:00.

**Figure 12:**
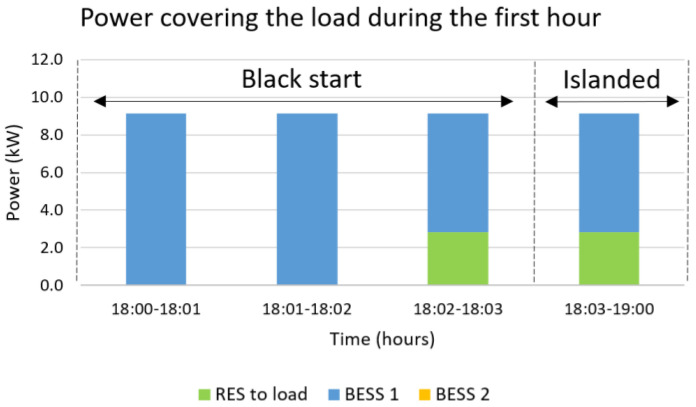
Power covering the load during the first hour for a black start at 18:00 followed by islanded operation until 21:00.

**Figure 13:**
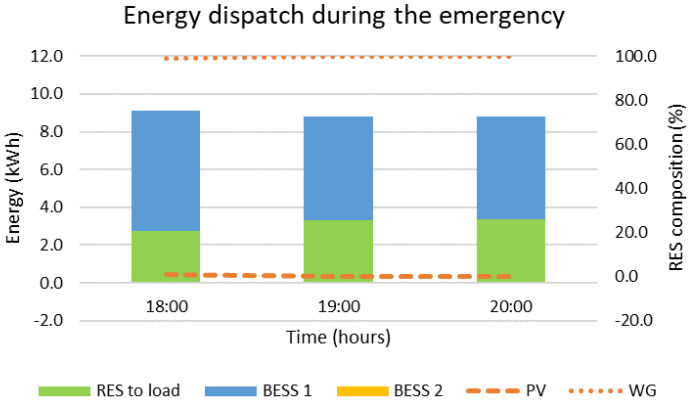
Energy dispatch for a black start at 18:00 followed by islanded operation until 21:00.

The blackout is considered to happen at 18:00, which is the worst possible scenario due to the maximized difference between the load and RES production, equal to 6.3 kW, followed by a three-hour islanded operation mode. The solution to this problem requires two timescales, a short one for the black start with time-steps set equal to one minute and a long one for the islanded mode with time-steps set equal to one hour. The results are presented in Figure 10, Figure 11, Figure 12 and Figure 13. During the first step of the black start, the two BESS are activated, since they constitute the only controllable supply units. Their activation is followed by the PVs in the second step, the production of which is close to zero due to the evening hour of the day, and the WG in the third step, the production power of which is equal to 2.8 kW. Afterwards, the MG operates islanded, as presented in Figure 12 and Figure 13, supported mostly by the main BESS, i.e., 64.8%, but also the WG, i.e., 35.2%. The overall restoration process requires the decrease of the main BESS’s state of charge by 56.8%. It is highlighted that, since the main BESS is charged with enough energy, the second BESS is activated but not utilized. As in the previous cases, so in this one, no RES curtailments are required, proving the correct dimensioning of the generation/storage assets of the MG. The most significant results are included in Table 3, below, along with the respective results for the rest of the simulated emergency cases.

**Table 3:** Main results from each simulated emergency case.

	Case 1	Case 2	Case 3
RES covering the load	100%	83.2%	35.2%
Main RES	PV	PV	WG
BESS covering the load (both, if connected)	0%	16.8%	64.8%
Overall SOC reduction for BESS 1	−9.3%	26.2%	56.8%
Overall SOC reduction for BESS 2	N/A	−9.0%	0%

Taking into account the results of all the aforementioned cases, it can be concluded that the abstract form of the optimizer, which constitutes the core of the proposed DSS, can serve a variety of emergencies with multiple decision variables, granting not only optimization but also a uniform approach. Furthermore, the results showcase the importance of having diverse and adequate RES profiles, which can support the environmentally friendly operation of the MG throughout the day, as well as the paramount necessity of adequate storage systems that ensure the MG’s autonomy.

## 5. Conclusions

This paper proposes an optimal and holistic DSS for emergencies occurring in MGs, which is considered to be a valuable service for the MG’s operator. The core of the DSS comprises a flexible optimizer that adapts to the nature of the emergency and aims at the maximization of the MG’s self-sufficiency, with special attention to the RES production, verified with the use of PowerFactory, DIgSILENT. The proposed system is tested on a MG that faces: (i) partial disconnection of the power supply, (ii) temporary disconnection from the main grid and (iii) a blackout. The results showcase the applicability and efficiency of the developed solution. 

## Figures and Tables

**Table 1:** MG description.

Component	Nominal Values
PV	Installed power: 15 kW
	Daily production for simulated day: 71.39 kWh
WG	Installed power: 5 kW
	Daily production for simulated day: 40.08 kWh
BESS 1	Usable energy: 50 kWh
	Nominal power: 15 kW
	Main BESS: yes
BESS 2	Usable energy: 15 kWh
	Nominal power: 5 kW
	Main BESS: no
Load	Installed load: 12 kW
	Daily demand: 162.08 kWh

**Table 2:** Description of emergencies.

	Case 1	Case 2	Case 3
Emergency description	Disconnection of BESS 2	Malfunction in the main grid	Blackout
Solution	Modified dispatch, without BESS 2	Islanded operation	Black start
Mode	Grid-following	Grid-forming	Grid-forming
Duration	Three hours	Six hours	Three hours
Interval	13:00–16:00	13:00–19:00	18:00–21:00

**Figure A1:**
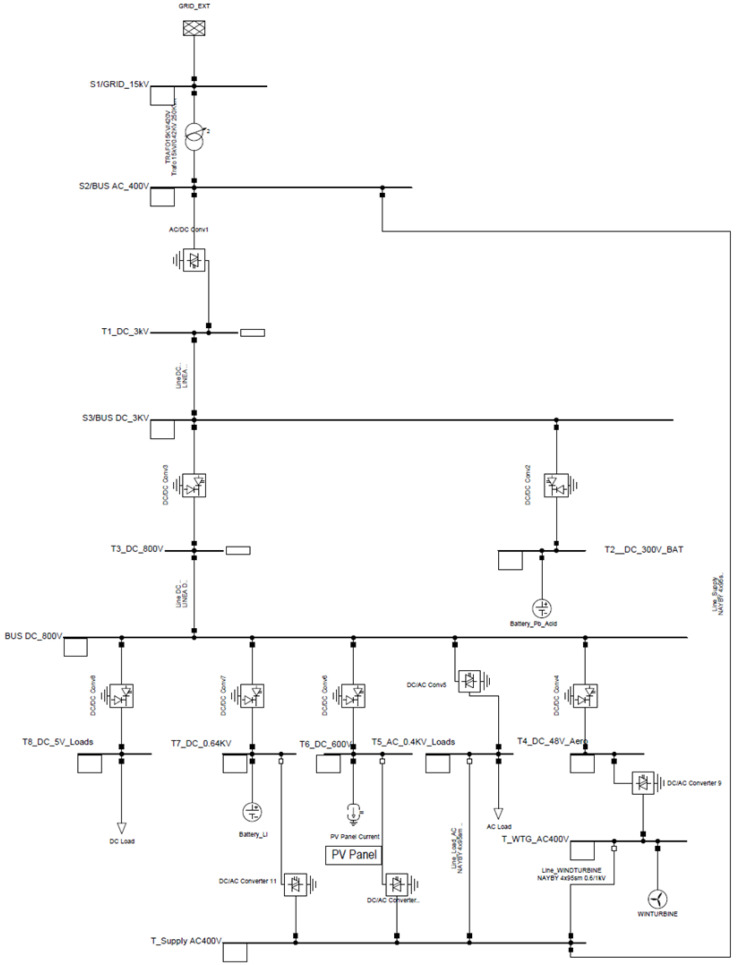
Set-up of the MG in PowerFactory, DIgSILENT.

## Data Availability

Not applicable.

## Appendix A

The set-up of the MG in PowerFactory, DIgSILENT is presented in Figure A1. It needs to be noted that the AC line on the right side of the topology, which connects the two LV AC nodes, is disconnected in the context of the TIGON project.
